# Origin of Degradation Phenomenon under Drain Bias Stress for Oxide Thin Film Transistors using IGZO and IGO Channel Layers

**DOI:** 10.1038/srep07884

**Published:** 2015-01-20

**Authors:** Jun Yong Bak, Youngho Kang, Shinhyuk Yang, Ho-Jun Ryu, Chi-Sun Hwang, Seungwu Han, Sung-Min Yoon

**Affiliations:** 1 Department of Advanced Materials Engineering for Information and Electronics, Kyung Hee University, Yongin-si, 446-701, Korea; 2 Department of Materials Science and Engineering and Research Institute of Advanced Materials, Seoul National University, Seoul 151-755, Korea; 3 Electronics & Telecommunications Research Institute (ETRI), Daejeon 305-350, Korea

## Abstract

Top-gate structured thin film transistors (TFTs) using In-Ga-Zn-O (IGZO) and In-Ga-O (IGO) channel compositions were investigated to reveal a feasible origin for degradation phenomenon under drain bias stress (DBS). DBS-driven instability in terms of V_TH_ shift, deviation of the SS value, and increase in the on-state current were detected only for the IGZO-TFT, in contrast to the IGO-TFT, which did not demonstrate V_TH_ shift. These behaviors were visually confirmed via nanoscale transmission electron microscopy and energy-dispersive x-ray spectroscopy observations. To understand the degradation mechanism, we performed *ab initio* molecular dynamic simulations on the liquid phases of IGZO and IGO. The diffusivities of Ga and In atoms were enhanced in IGZO, confirming the degradation mechanism to be increased atomic diffusion.

Recent issues regarding oxide TFTs have focused on improving their long-term stability under various stress conditions, such as gate-bias stress^1^, light illumination stress under gate bias^2^, and the ambient aging effect^3^. Among these, threshold voltage (V_TH_) instability under the NBIS condition is one of the most critical issues for realizing the active-matrix backplane composed of oxide TFTs. To solve this problem, research involving viewpoints of channel layer compositions^4^, structure^5^, and preparation conditions^6^ has occurred. Despite recent improvements in NBIS, instability still remains a major challenge for oxide TFTs.

On the other hand, the instability characteristics under drain-bias stress (DBS) conditions should also be carefully investigated^7^. When oxide TFTs are used as driver devices for active-matrix organic lighting-emitting diode (AMOLED) pixels, the TFTs operate in a saturation mode to supply a stable constant current to the OLED and, hence, may experience higher DBS during operation^8^. Furthermore, in order to employ oxide TFTs as a switch device in a gate driver, higher stability under large drain bias and a wide swing range of drain voltages should be guaranteed for circuit operations.

From our previous report^9^, the choice of electrode materials was found to be key in securing the reliability of In-Ga-Zn-O (IGZO) TFTs under DBS. However, a detailed degradation mechanism for long-term device reliability under the DBS was not clearly established. Furthermore, in-depth studies on DBS stability are necessary to determine its origin.

This study focused on the effect that channel material has on the DBS stability. The specific channel compositions chosen were IGZO and In-Ga-O (IGO). It is generally accepted that IGZO is the most favorable material for oxide TFT applications owing to several advantages, including high mobility, wide process window, and superior stability. The In_2_O_3_ incorporated with suitable amounts of Ga can also be expected to be one of the promising compositions to achieve both high mobility and operational stability^10^. The two different TFTs fabricated using the IGZO and IGO channels (termed IGZO-TFT and IGO-TFT) were compared from the viewpoint of variations in the DBS stabilities. Nanoscale observations of interfacial regions were performed to provide visual evidence. In order to elucidate the microscopic origin of the DBS degradation phenomenon, the physical chemical behaviors for each channel compositions were carefully investigated.

## Results

**Figure 1:**
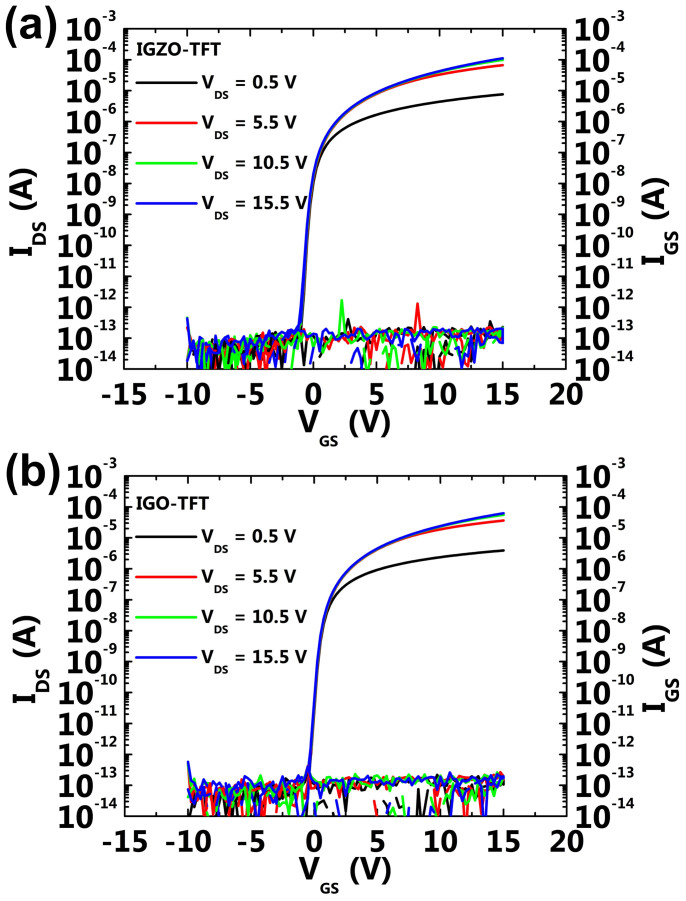
I_DG_−V_GS_ transfer characteristics of (a) IGZO-TFT and (b) IGO-TFT. The measurements were performed in a double sweep mode of V_GS_ at V_DS_'s of 0.5, 5.5, 10.5, and 15.5 V. The channel width and length of the measured devices were defined as 40 and 20 μm, respectively. The final annealing temperatures for IGZO and IGO-TFTs were 250 and 300°C.

Figures 1(a) and (b) show the drain current–gate voltage (I_DS_-V_GS_) characteristics of the fabricated IGZO and IGO TFTs, respectively. The estimated device parameters such as saturation mobility (μ_sat_), subthreshold-swing (SS), and threshold voltage (V_TH_) for the IGZO and IGO TFTs were obtained and found to be 14.8 cm^2^ V^−1^ s^−1^, 210 mV decade^−1^, and 1.8 V and 6.5 cm^2^ V^−1^ s^−1^, 212 mV decade^−1^, and 1.7 V, respectively. There were no remarkable differences including the sufficient low gate leakage current (I_GS_) of lower than 10^−12^ A between the two devices except for the saturation mobility. Next, variations in the transfer characteristics of the IGZO and IGO TFTs were investigated during gate-bias stress conditions with V_GS_ of −20 V and +20 V for 10^4^ s, as shown in Figure S1. The transfer curves for each TFT were measured as the lapse of stress time at a fixed drain voltage (V_DS_) of 10 V. As can be seen in the figures, both IGZO and IGO TFTs demonstrated stable operation with only a small degree of variation in V_TH_ shifts. The strong endurance against gate-bias stress might have originated from the dense Al_2_O_3_ gate insulator and the protection layer prepared to prevent chemical and mechanical damage to active channels^11^. From these results, the properties regarding stability for both TFTs with different active channels of IGZO and IGO were confirmed under positive and negative bias stress conditions even after a lapse of stress time of 10^4^ s.

**Figure 2:**
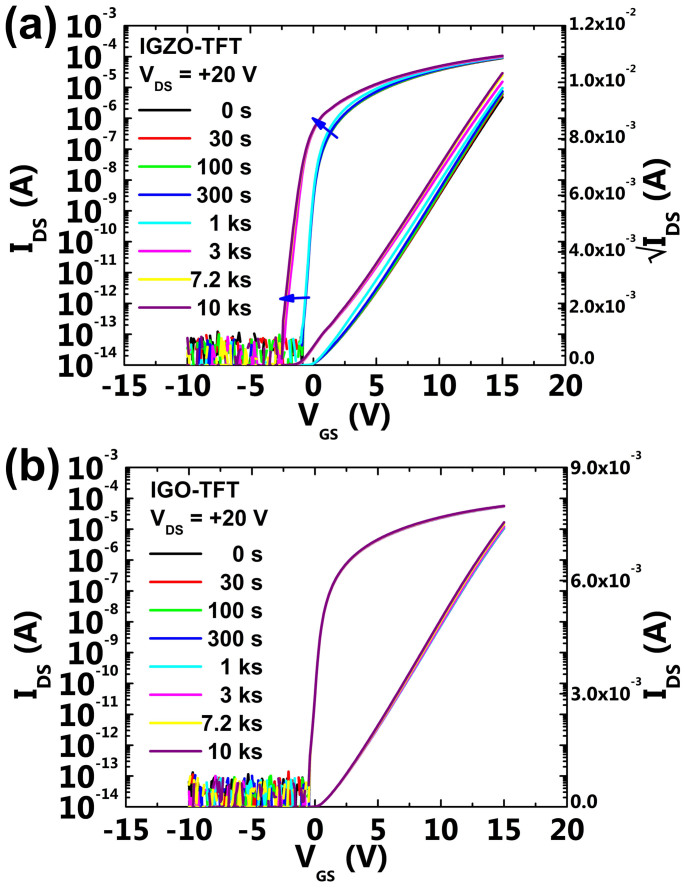
Variation in the I_DG_−V_GS_ transfer characteristics for the (a) IGZO-TFT and (b) IGO-TFT with a lapse of stress time for 10^4^ s under the DBS of 20 V. A V_DS_ of 10 V was applied for the measurements. The negative shift in V_TH_ for the IGZO-TFT was 1.7 V after the DBS, contrary to the IGO-TFT, which showed almost no shift in V_TH_.

Marked differences between the IGZO and IGO TFTs appeared when the devices were characterized under DBS conditions, in which a V_DS_ of 20 V was continuously applied to the ITO drain electrode of IGZO and IGO TFTs for 10^4^ s, as shown in Figures 2(a) and (b), respectively. During the DBS test, the values of μ_sat_, SS, and V_TH_ for the IGZO-TFT varied from 11.8 cm^2^ V^−1^ s^−1^, 191 mV decade^−1^, and 1.4 V to 12.1 cm^2^ V^−1^ s^−1^, 243 mV decade^−1^, −0.3 V, respectively. The degradation of SS and negative shifts in V_TH_ were observed. It was also noticed that the derivatives of I_DS_ in the vicinity of the saturation region with respect to V_GS_ decreased according to an increase in stress time. On the other hand, for the IGO-TFT, the device parameters did not undergo any significant changes during the DBS tests for the same stress duration. Values of μ_sat_, SS, and V_TH_ before and after the DBS tests were estimated to be 5.2 cm^2^ V^−1^ s^−1^, 221 mV decade^−1^, and 0.8 V and 5.3 cm^2^ V^−1^ s^−1^, 193 mV decade^−1^, and 0.8 V, respectively. From this series of evaluations of the transfer characteristics, it was confirmed that the IGO-TFT exhibited excellent DBS stability compared to those of the IGZO-TFT. From our previous reported work^9^, the effects of electrode materials on the device stabilities under DBS condition were investigated. Via nanoscale TEM images and EDS analysis, we could found that the Ti device annealed at a higher temperature exhibited an enhanced stability under the DBS due to the synergy effects of robust interface and role of transition layer acting as a barrier contrary to the severely degraded ITO device after the DBS. Considering these results, if the increased carrier concentration of whole IGZO bulk was a main factor for the degradation under DBS, the Ti device should also be degraded under the DBS condition of 20 V. The increased carrier in IGZO bulk can be ruled out as a main origin for the device degradation after the DBS.

**Figure 3:**
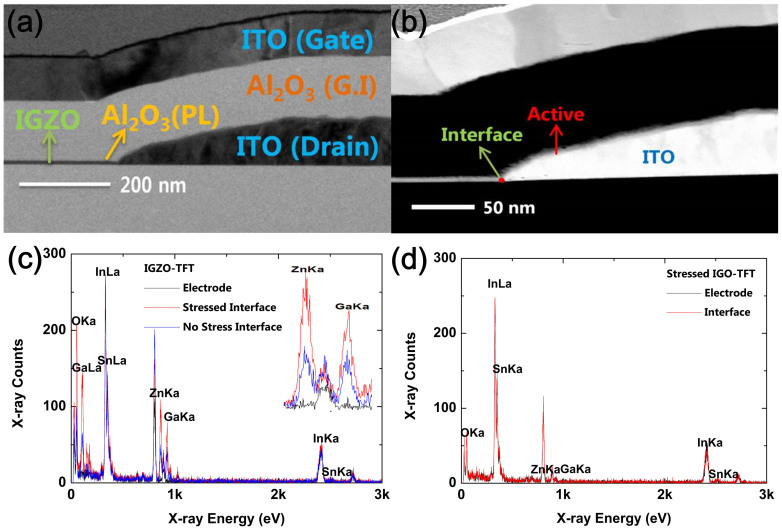
Cross-sectional TEM views of (a) bright and (b) STEM-HAADF images of the interfacial region between the drain electrode and channel layer for the IGZO-TFT. EDS spectra at the ITO electrode and interfacial regions, which were defined as an electrode area near the channel layer, (c) for the IGZO-TFT and (d) IGO-TFT before and after the DBS.

To verify these remarkable differences between the devices in terms of the DBS instability, the interface regions between the drain electrode and IGZO or IGO channel layers were observed by means of transmission electron microscopy (TEM). Figures 3(a) and (b) show bright TEM and scanning TEM (STEM)-high angular annular dark-field (HAADF) images for the IGZO-TFT, respectively. The device was confirmed to be suitably fabricated with each part of the IGZO channel, Al_2_O_3_ gate insulator, and ITO drain and gate electrodes, as designed. Although it was hard to recognize the domains between the channel and drain electrodes from the STEM image shown in Figure 3(b) due to the same element of In in both IGZO and ITO, their components as well as accurate component locations could be discerned through the STEM-EDS mapping images shown in Figure S2. TEM and STEM-HAADF images for the IGO-TFT were also shown in Figure S3. For a quantitative analysis of the interatomic diffusion near the interfaces, point energy-dispersive x-ray spectroscopy (EDS) measurements were performed at areas within the ITO electrode just next to the IGZO or IGO channel layers, as shown in Figures 3(c) and (d), respectively. In addition, the EDS results including the bulk channel region for IGZO and IGO TFTs were shown in Figure S4. For IGZO-TFT, all constituents of In, Ga, Zn, and Sn were simultaneously detected in the interfacial region after the application of DBS for 10^4^ s, which was completely different from the results of the same device before the DBS test. It was especially noticeable that the magnitude of Ga and Zn peaks after the DBS definitely increased, as described in the inset of Figure 3(c). This phenomenon strongly suggested that the considerable interdiffusion between the IGZO and ITO was caused by the application of DBS. Under strong drain bias applied for a long time, the temperature of the specified region near the interface would increase due to self-heating effects, as confirmed in the previous literatures^11^,^12^. This may be the primary driving force accelerating the diffusion process from the concentration gradient of constituent elements between the drain electrode and channel regions. Additionally, the interface damaged by the ion bombardment during the channel deposition process could be suggested to accelerate undesirable interdiffusion, even though it would have minor impact^13^,^14^. This DBS-driven interdiffusion might induce some compositional changes at the interface close to the drain electrode^9^. The carrier concentration at specified regions near the drain electrode happened to increase by the compositional change and/or atomic rearrangement. Eventually, localized more conductive regions might influence the negative shift in V_TH_ and the increase in on-state current at the saturation region by the percolation effect. On the other hand, for the IGO-TFT, the Ga and Zn peaks observed at the interfacial region were nearly identical before and after the DBS test for 10^4^ s. Consequently, the IGO channel was thought to be a more robust material compared with the IGZO channel against harsh DBS conditions. For the interface of the source side after DBS measurements, the TEM, STEM and EDS analysis were conducted to find out whether the interface of the source side was degraded or not. Figure S5 shows the TEM, STEM images, and the results of the point EDS for the IGZO-TFT at the source side after DBS measurement. As shown in Figure S5(c), the interface between the channel and electrode of source side did not experience severe interdiffusion phenomenon between the constituent atoms of In, Ga, and Zn, which was totally different from the results of point EDS at the drain-side interface. Moreover, when comparing the EDS results between the source electrode and the interface, as shown in Figure S5(d), there were no marked differences in levels of peaks for In, Ga, Zn, and Sn, except for the oxygen. From these results in two perspectives, the source-side interface was not severely affected by DBS measurements. In addition, considering these results, the SS value degradation of IGZO-TFT after the DBS can be explained by defect creation model^15^,^16^,^17^,^18^. Occasionally, SS value degradation in company with negative V_TH_ shifts and fluctuated drain currents could be caused by various stress conditions such as bias, light illumination stresses and adsorption and/or desorption oxygen. The additional defects such as cation vacancies and/or oxygen vacancies could be created when the inter-diffusion process between the constituent elements at specified region between IGZO channel and ITO electrode was accelerated by high DBS.

**Figure 4:**
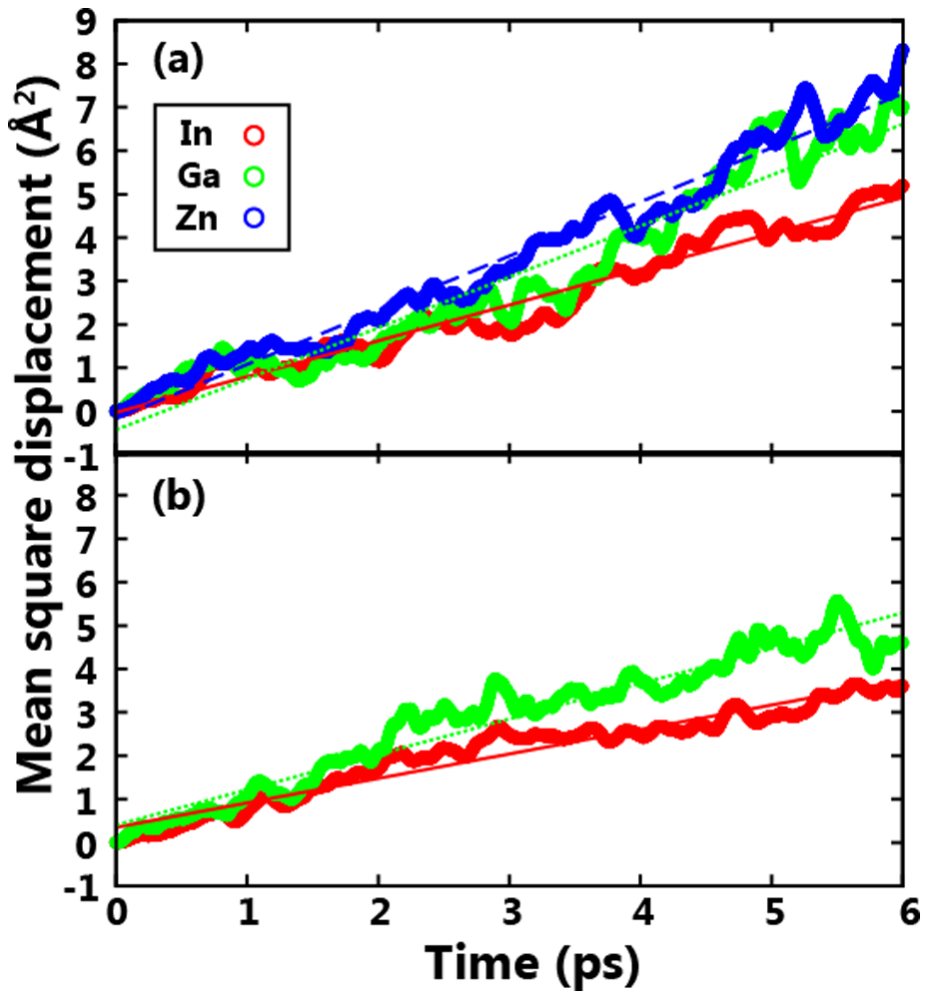
Calculated mean square displacements for liquid phases of (a) IGZO (In:Ga:Zn = 2:1:2) and (b) IGO (In:Ga = 2:1) during MD simulations at 2000 K.

To verify the above assumption that atomic diffusion was enhanced in IGZO compared to IGO, an *ab initio* molecular dynamics (MD) simulation was carried out for the two materials. Since the atomic diffusion process in the solid state is too slow to be observed within a feasible simulation time, we studied the diffusion coefficient (*D*) of cations in the liquid state to provide indirect evidence. To this end, the MD simulations at 2000 K were performed for each amorphous structure for 6 ps. The mean square displacements (MSD) were calculated for each metal type as shown in Figure 4 and *D* was obtained by a linear regression of the MSD data. For the IGZO channel, the values of *D* for In, Ga, and Zn were found to be 2.23 × 10^−6^, 2.63 × 10^−6^, and 4.54 × 10^−6^ m^2^ s^−1^, respectively, while those of In and Ga in the IGO channel were estimated to be 7.65 × 10^−5^ and 1.18 × 10^−6^ m^2^ s^−1^, respectively. There were marked differences in *D* of In and Ga between IGZO and IGO. Since the atomic ratio of In/Ga was the same for the two compositions, the enhanced *D* values in IGZO could be attributed to the Zn atoms. The smaller coordination number for Zn than that for either In and Ga may reduce the migration barriers of cations. The increased diffusivities in IGZO were consistent with the above conjecture that a significant change in the density of Zn and Ga in IGZO after DBS originated from interdiffusion driven by local heating. To clearly verify the origins of DBS instability caused by the self-heating effect and the high diffusivity for the IGZO channel, the IGZO and IGO TFTs were also evaluated under the DBS conditions of 10, 20, and 40 V (IGZO-TFT) and 40 V, and 60 V (IGO-TFT). As shown in Figure S6, the estimated negative shifts in V_TH_ for the IGZO-TFT after the DBS tests at 10, 20, and 40 V were approximately 0, 1.0, and 1.9 V, respectively. By increasing the drain bias stress from 20 to 40 V, the negative shifts in V_TH_ and the increases in I_DS_ were accelerated. On the other hand, the IGO-TFT was definitely stable even after the DBS test at 40 V and 60 V thanks to lower diffusivity of IGO channel. In addition, if hot-carrier effects were dominated for the fabricated two devices, the negative shifts in V_TH_ under the DBS must be observed in IGO device with the same gate length as the IGZO device. When considering the differences in device mobility for each TFT as a main factor for the degradation phenomenon under DBS, the IGO TFT should be degraded under DBS measurement with 60 V condition which was three times higher than normal DBS condition of 20 V. Moreover, the stability characteristics of IGZO TFT was also examined with the combination of V_DS_ and V_GS_ of 20 and 10 V, as shown in Figure S7. There was no V_TH_ shift in any directions. This interesting behavior can be interpreted with the reduction of the lateral drain bias effect, which was induced by the vertical electric field from the gate bias. From these obtained results, the self-heating effect and interdiffusion phenomenon originated from channel composition near the drain electrode strongly supported the DBS instability of the IGZO TFT as one of the main origins.

## Discussion

It was confirmed that DBS-driven instabilities were strongly related to the oxide channel compositions of IGZO and IGO. When DBS was applied for 3 h, a marked negative shift in V_TH_ and increase in the SS value and the on-state currents were observed for only IGZO-TFT, in contrast to IGO-TFT, which did not display any degradation in terms of device performance. This instability phenomenon was verified by nanoscale TEM and EDS observations, in which a higher degree of interdiffusion of the constituent elements was confirmed for IGZO, compared with the IGO channel. The MD simulation results support the fact that the atomic interdiffusion between the drain electrode and channel was severely activated for the IGZO channel owing to the role of Zn, which could be a primary reason for DBS instability. Consequently, in order to guarantee device stability during DBS, channel composition should be carefully optimized in terms of atomic diffusion phenomena.

## Methods

**Figure 5:**
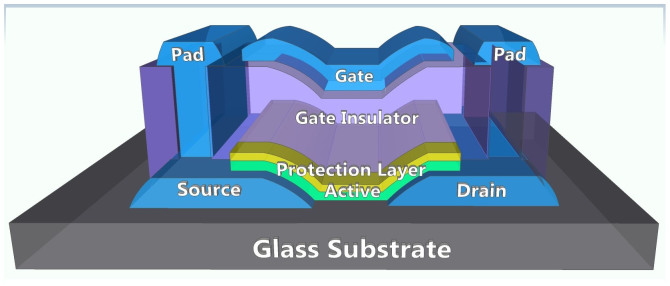
Schematic diagram of fabricated top-gate structured TFTs.

Two transparent oxide TFTs employing different channel materials of IGZO and IGO were fabricated with a top-gate bottom-contact structure, as shown in Figure 5. The 150-nm-thick ITO was deposited on the glass substrate as S/D electrodes. The 20-nm-thick channel layers of IGZO (In:Ga:Zn = 2:1:2 atomic ratio) and IGO (In:Ga = 2:1 atomic ratio) were separately prepared by an rf sputtering method. In order to prevent chemical and mechanical damage during the patterning process^19^, the 9-nm-thick Al_2_O_3_ layer was formed by atomic layer deposition (ALD) at 200°C. Then, Al_2_O_3_ gate insulators were formed by ALD at 150°C. Although their thickness values were not identical, which were 176 and 120 nm for the IGZO and IGO TFTs, respectively, the electrical evaluations were conducted considering this difference. All the patterning processes were performed by conventional photolithography and wet etching methods. The fabricated devices were annealed in a vacuum atmosphere at 250 and 300°C, respectively. The device characteristics were evaluated by a semiconductor parameter analyzer (Keithley 4200-SCS) in a dark box at room temperature. The channel width and length of measured devices were 40 and 20 μm, respectively.

The cross-sectional nanoscale microstructures for the neighborhood of drain electrode including channel area were observed using TEM (FEI TecnaiG^2^ F30 S-Twin) at an accelerating voltage of 300 kV. The compositions of each constituted layer were carefully investigated by using a STEM-HAADF and EDS. The observed TEM samples were prepared using a dual-beam focused ion beam (DB-FIB; FEI, Nova200) in TEM sample preparation mode. The *D* of each atom type was examined by first-principles calculations using the Vienna ab initio simulation package (VASP)^20^,^21^ with similar computational setups to those reported in Ref. 22 and 23. The first-principles MD simulations were performed for modeling amorphous structures of a-IGZO (In:Ga:Zn = 2:1:2) and a-IGO (In:Ga = 2:1). The six (eight) formula units of In_4_Ga_2_Zn_4_O_13_ (In_4_Ga_2_O_9_) for a-IGZO (a-IGO) were distributed randomly into cubic supercells. They were heated to 3,000 K for 10 ps, and then, quenched to 300 K at a rate of −300 K/ps. The lattice parameters and atomic positions were fully optimized until residual force action on each atom was less than 0.02 eV/Å.

## Author Contributions

S.Y., H.J.R., C.S.H. and J.Y.B. designed this work. J.Y.B. fabricated the devices and measured the electrical characteristics. Y.K. and S.H. performed the theoretical calculations. J.Y.B., S.M.Y. and S.H. wrote the manuscript. All authors discussed the results and commented on the manuscript. The project was supervised by S.M.Y.

## Supplementary Material

Supplementary Information SREP-14-04516C_Supplementary Information
