# Supplementary material for: Origin of Degradation Phenomenon under Drain Bias Stress for Oxide Thin Film Transistors using IGZO and IGO Channel Layers
Source: Sci Rep. 2015 Jan 20;5:7884. doi: 10.1038/srep07884 (PMC4298721; doi:10.1038/srep07884)
Supplement: Supplementary Information — SREP-14-04516C_Supplementary Information [file srep07884-s1.doc]

**Supplementary Information**

**Origin of Degradation Phenomenon under Drain Bias Stress for Oxide Thin Film Transistors using IGZO and IGO Channel Layers**

Jun Yong Bak^1^, Youngho Kang^2^, Shinhyuk Yang^3^, Ho-Jun Ryu^3^, Chi-Sun Hwang^3^, Seungwu Han^2^, and Sung Min Yoon^1^,*

^1^Department of Advanced Materials Engineering for Information and Electronics, Kyung Hee University, Yongin-si, 446-701, Korea.

^2^Department of Materials Science and Engineering and Research Institute of Advanced Materials, Seoul National University, Seoul 151-755, Korea.

^3^Electronics & Telecommunications Research Institute (ETRI), Daejeon 305-350, Korea.

Correspondence and requests for materials should be addressed to S.M.Y ([**sungmin@khu.ac.kr**](mailto:sungmin@khu.ac.kr))


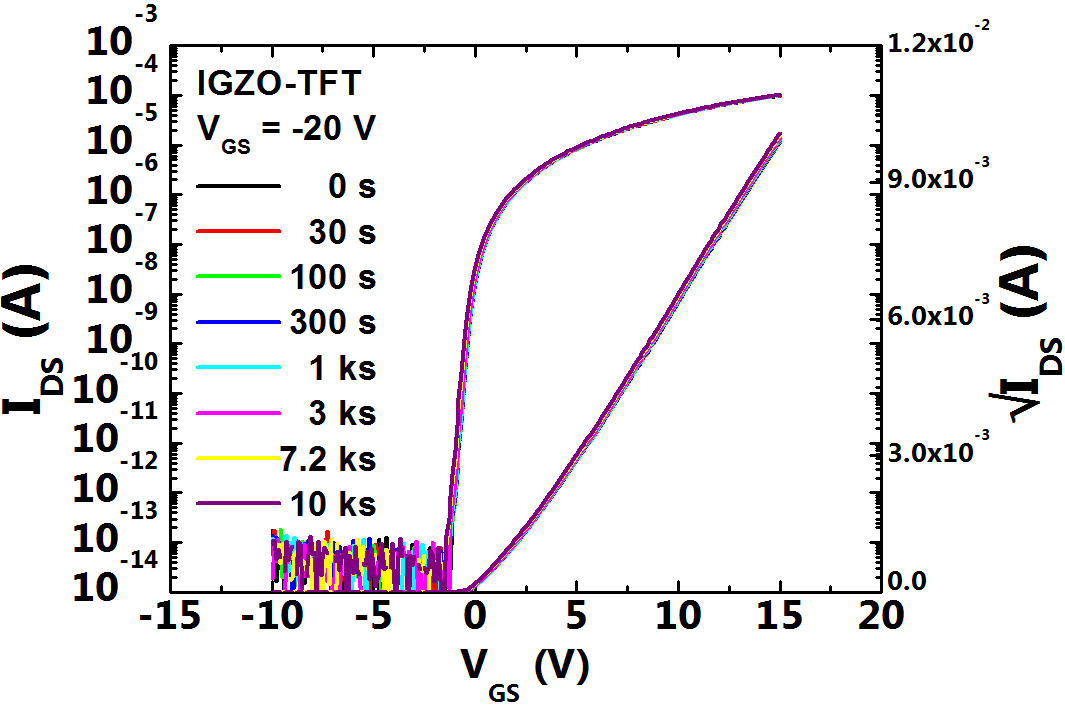

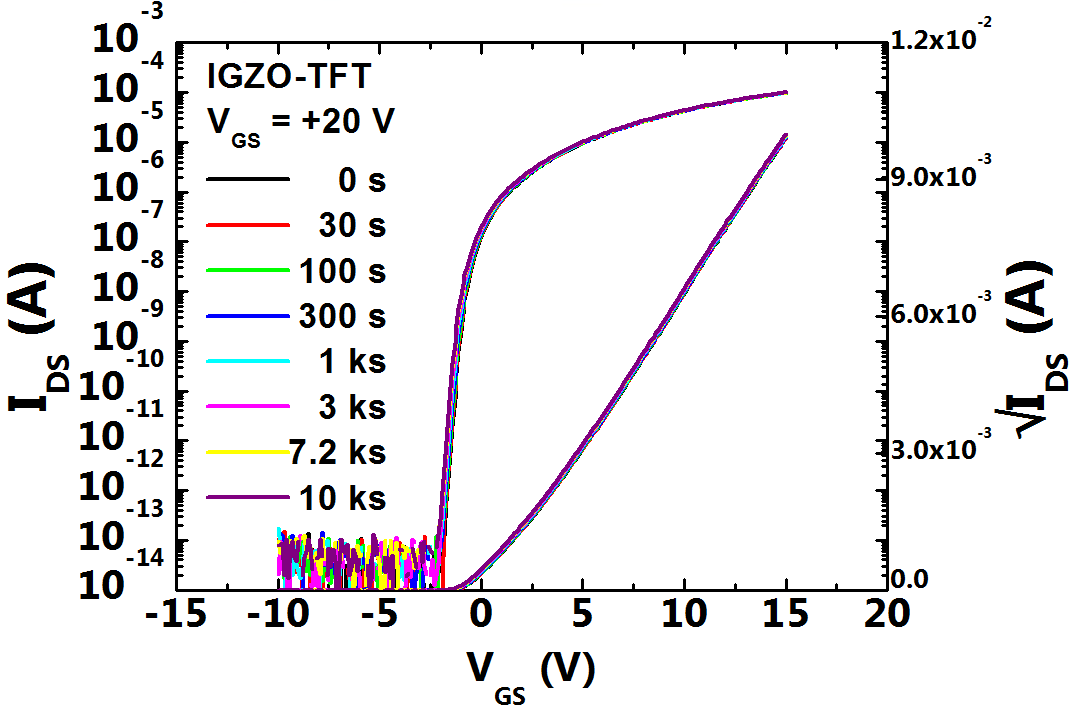


S1-(a) S1-(b)


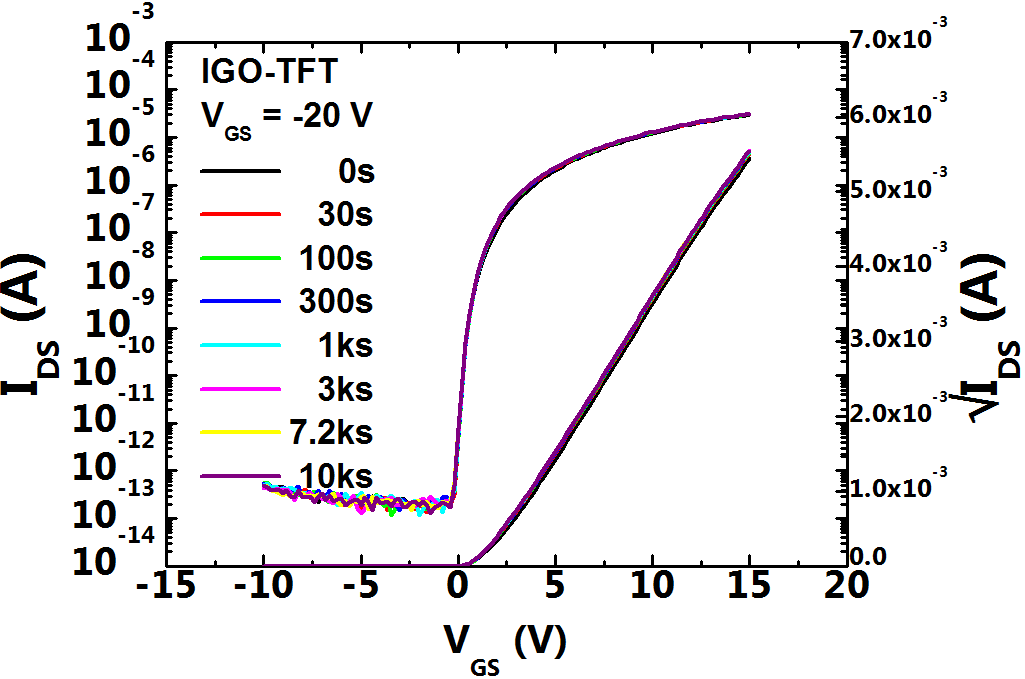

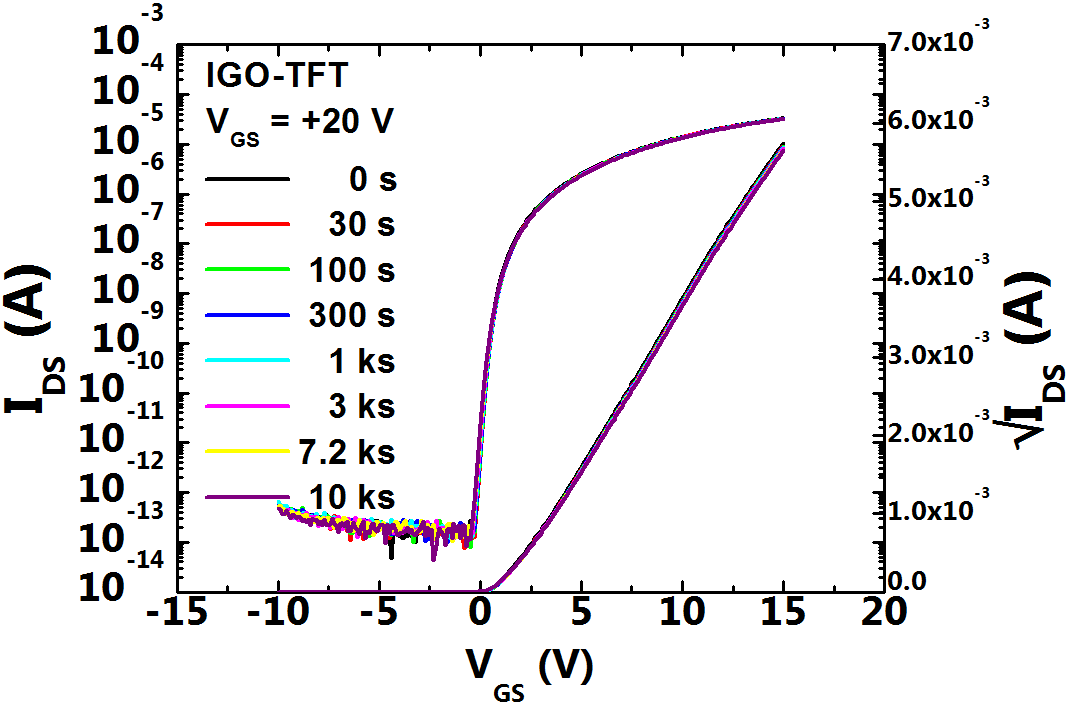


S1-(c) S1-(d)

**Figure S1.** Variation in the I_DG_−V_GS_ transfer characteristics for the IGZO and IGO-TFTs with respect to the stress time for 10^4^ s under gate bias stress conditions. The transfer curves for the IGZO and IGO-TFTs were measured at the applied V_GS_ of (a) −20 and (b) +20 V and at the application of V_GS_ of (c) −20 and (d) +20 V, respectively. V_DS_ of 10 V was fixed for the measurements. The negative shifts in V_TH_ for both devices were obtained to be lower than 0.1 V after the positive or negative gate-bias stress, respectively.


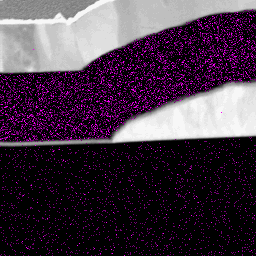

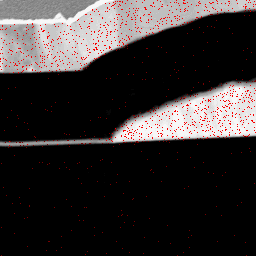


S2-(a) S2-(b)


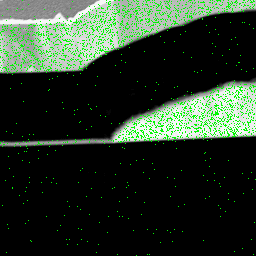

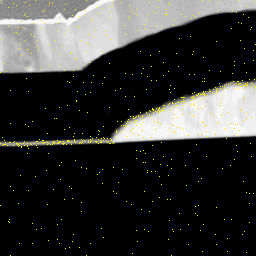


S2-(c) S2-(d)


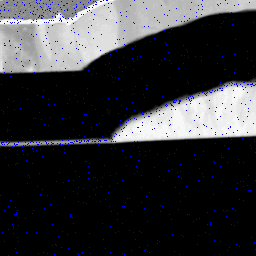

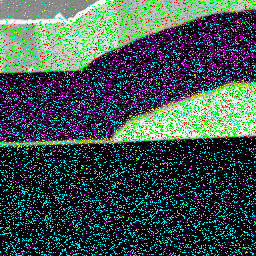


S2-(e) S2-(f)

**Figure S2.** EDS mapping images of the interface region between the drain electrode and IGZO channel layer for the IGZO-TFT corresponding to (a) Al (purple), (b) In (red), (c) Sn (green), (d) Zn (yellow), (e) Ga (blue), and (f) various compositions overlapped with Al, In, Sn, and Zn, including oxygen (cyan).


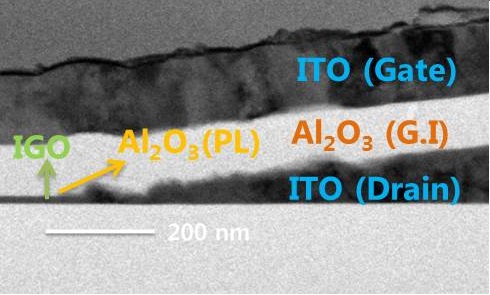

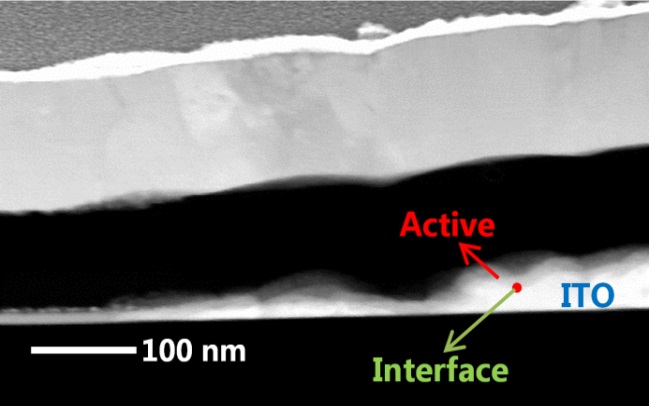


S3-(a) S3-(b)


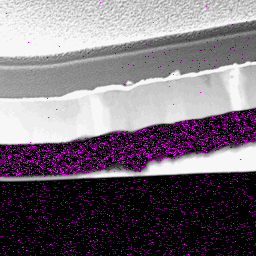

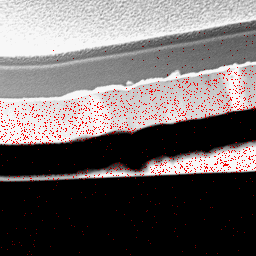


S3-(c) S3-(d)


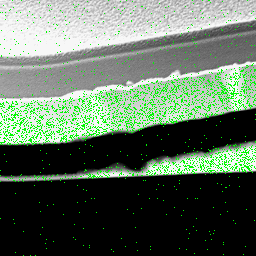

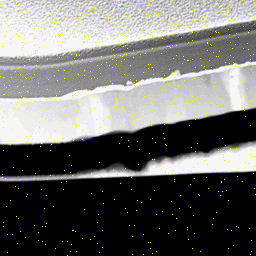


S3-(e) S3-(f)


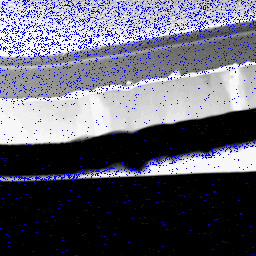

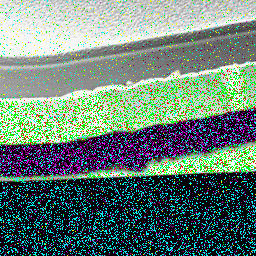


S3-(g) S3-(h)

**Figure S3.** Cross-sectional TEM views of (a) bright and (b) STEM-HAADF images of the interface region between the drain electrode and channel layer for the IGO-TFT. EDS mapping images of the interface region between the drain electrode and IGZO channel layer for the IGO-TFT corresponding to (c) Al (purple), (d) In (red), (e) Sn (green), (f) Zn (yellow), (g) Ga (blue), and (h) various compositions overlapped with Al, In, Sn, and Zn including oxygen (cyan).


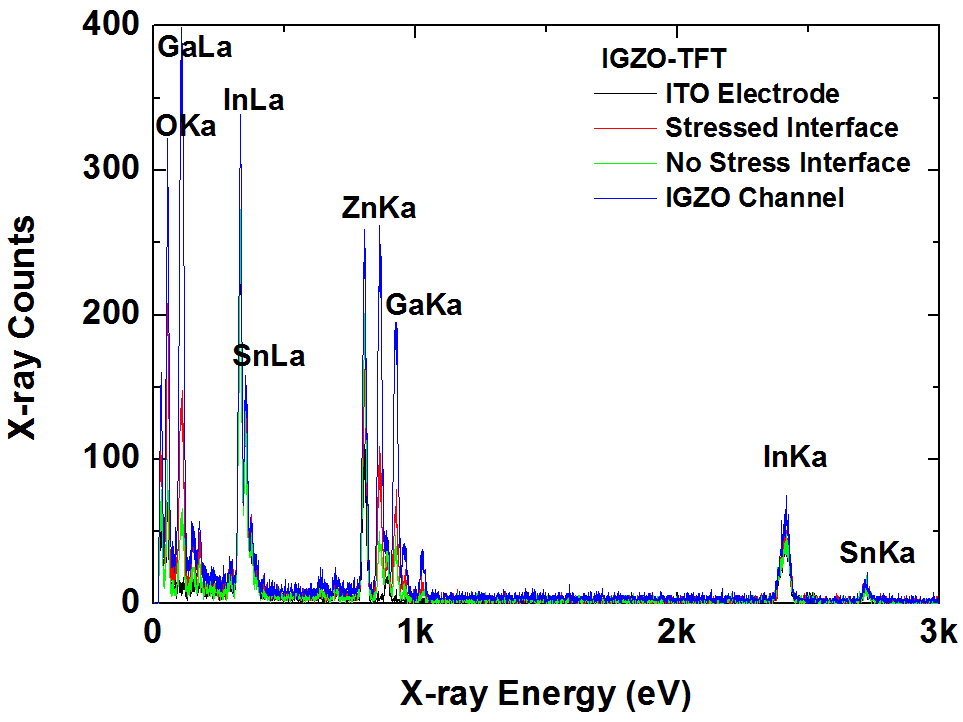


S4-(a)


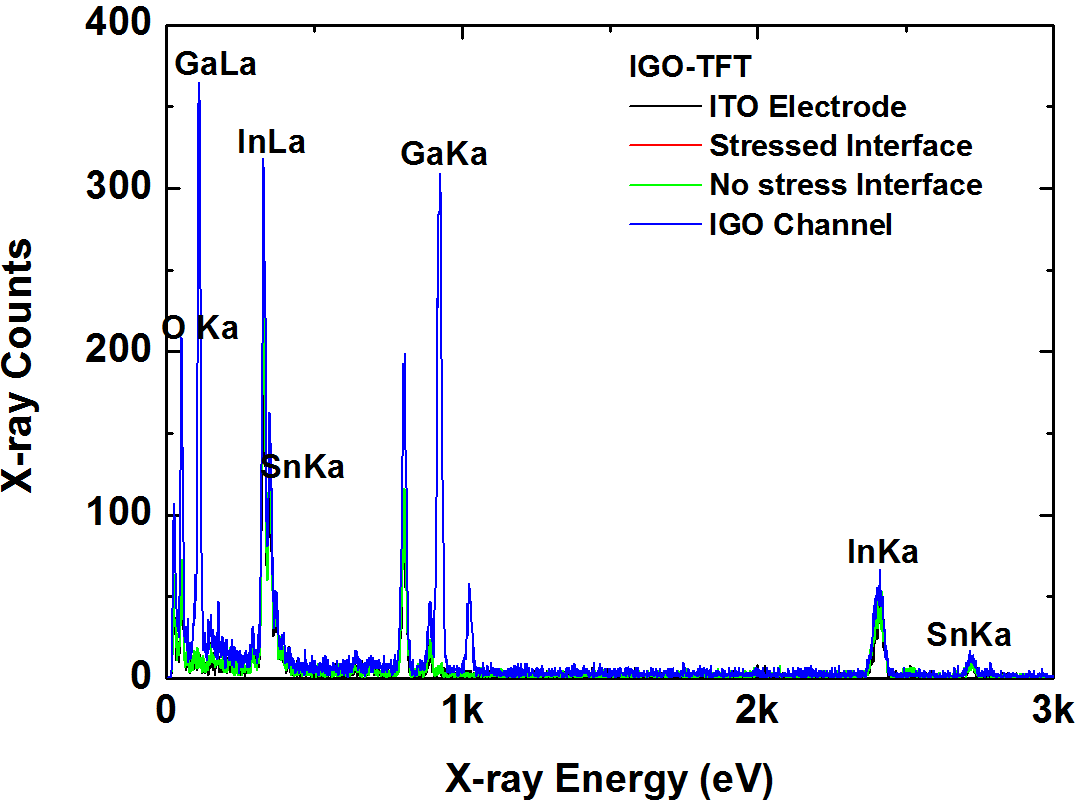


S4-(b)

**Figure S4.** EDS spectra at the bulk channel, ITO electrode and interface regions, which defined as an electrode area near the channel layer, for the (a) IGZO-TFT and (b) IGO-TFT before and after the DBS measurements.


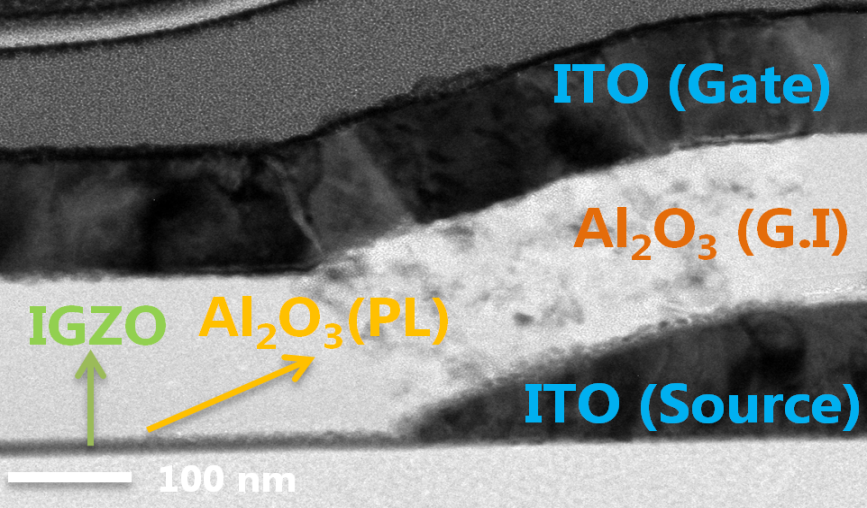

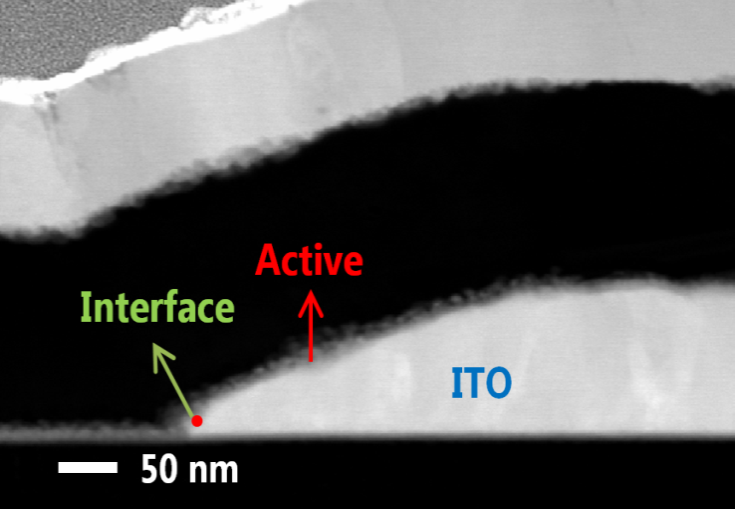


S5-(a) S5-(b)


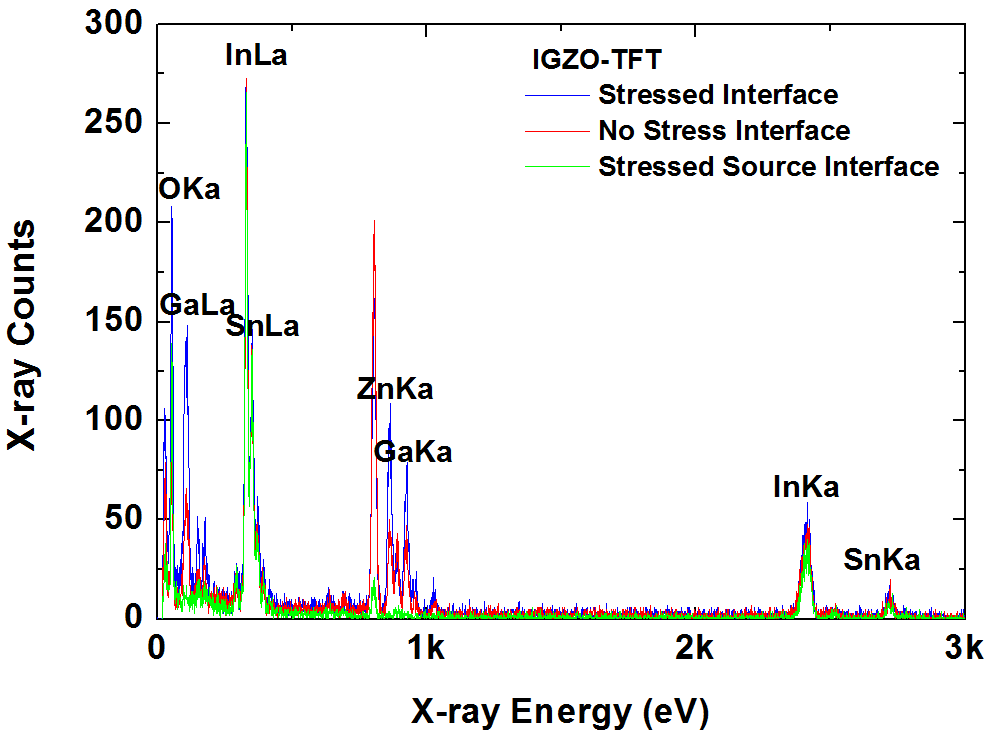

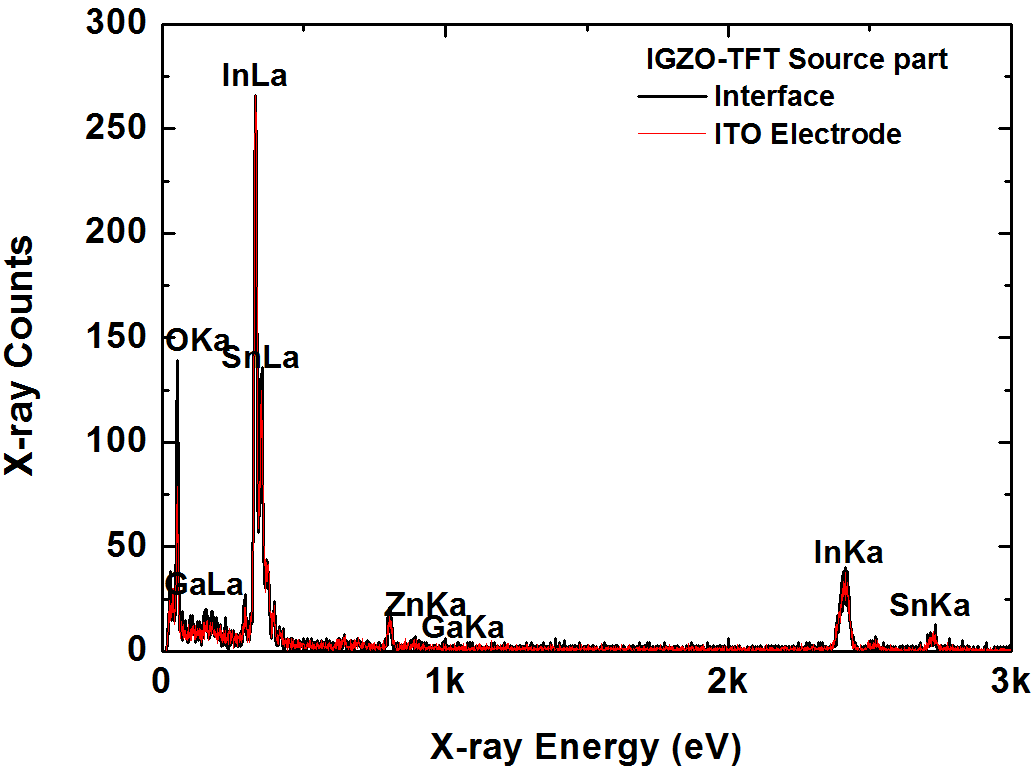


S5-(c) S5-(d)

**Figure S5.** Cross-sectional TEM views of (a) bright and (b) STEM-HAADF images of the interface region between the source electrode and channel layer for the IGZO-TFT. (c) EDS spectra at the interface regions, which defined as an electrode area near the channel layer, for the drain and source electrode of the IGZO-TFT before and after the DBS measurements. (d) EDS spectra at the source electrode and the interface regions for the source electrode of the IGZO-TFT after the DBS measurements.


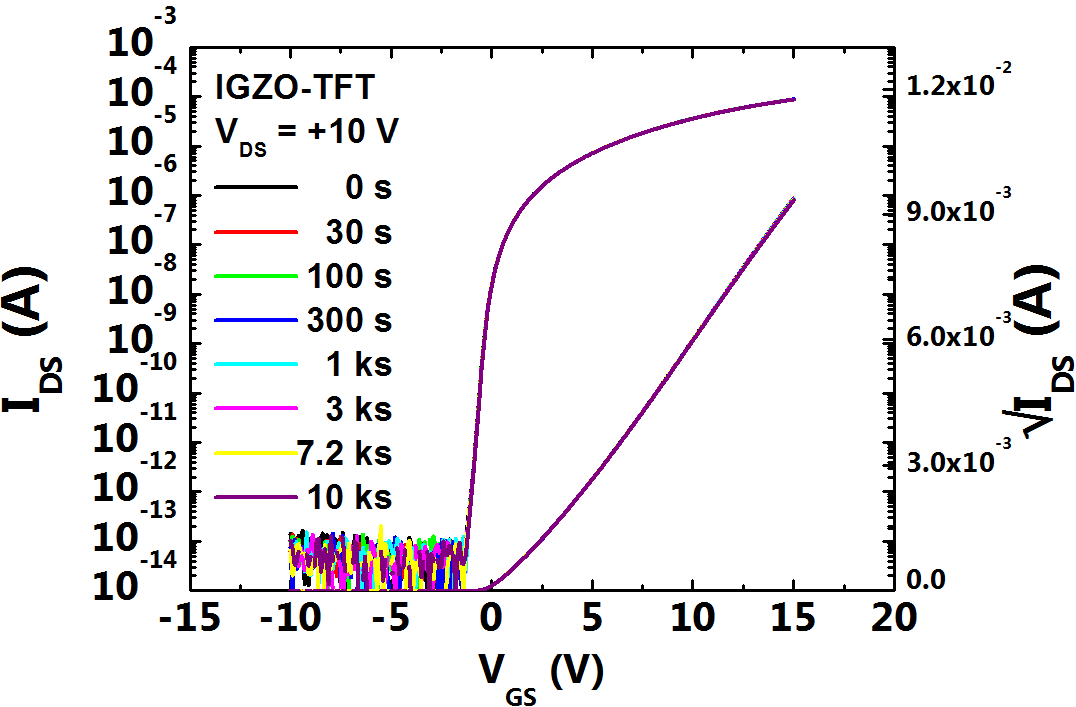

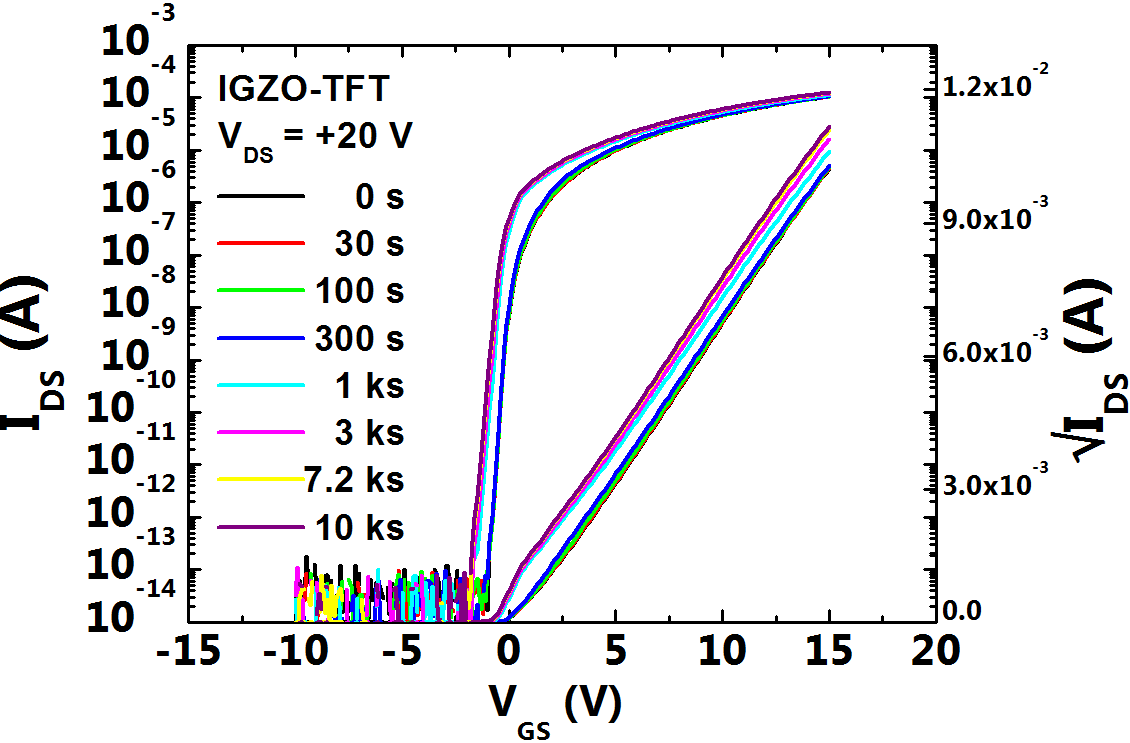


S6-(a) S6-(b)


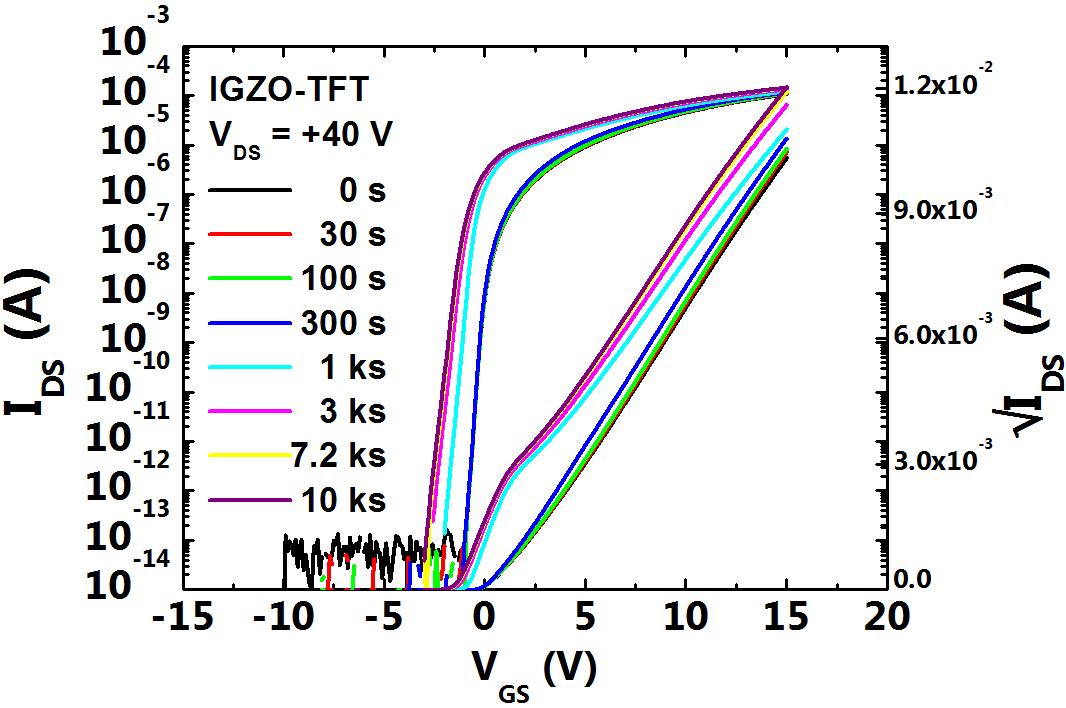

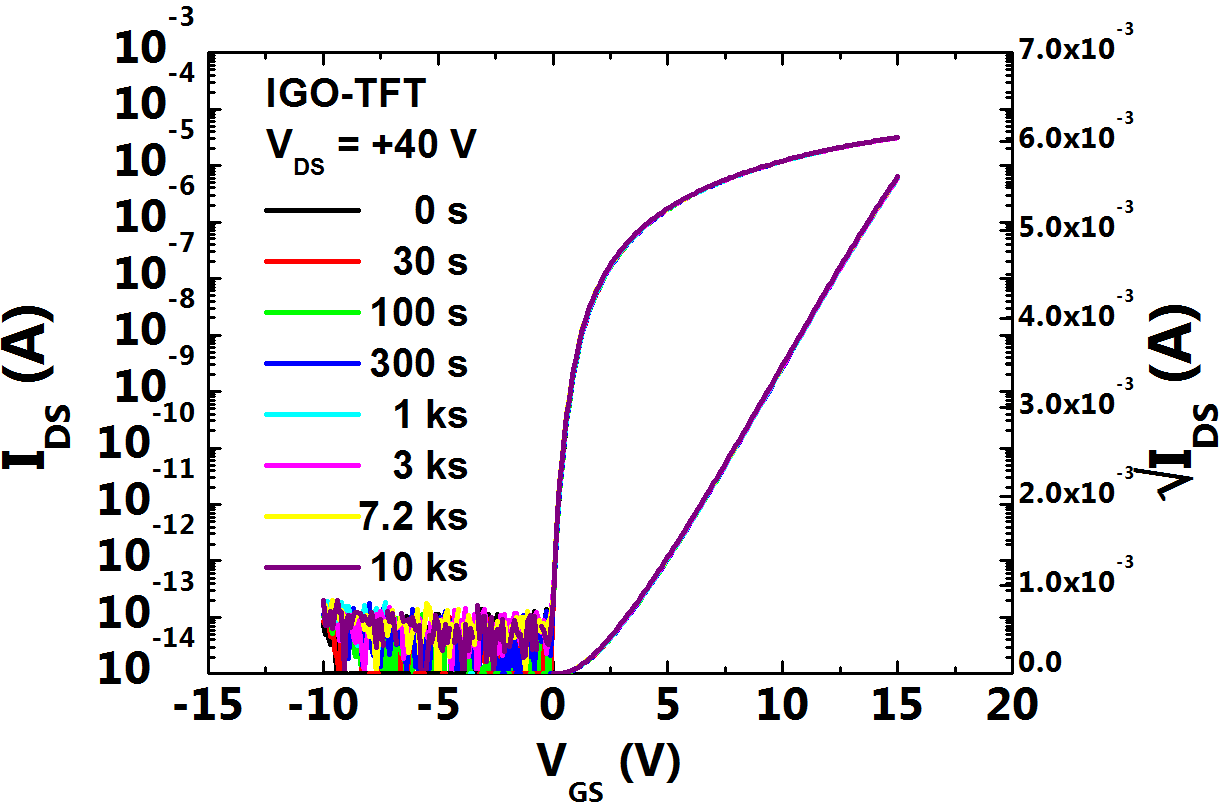


S6-(c) S6-(d)


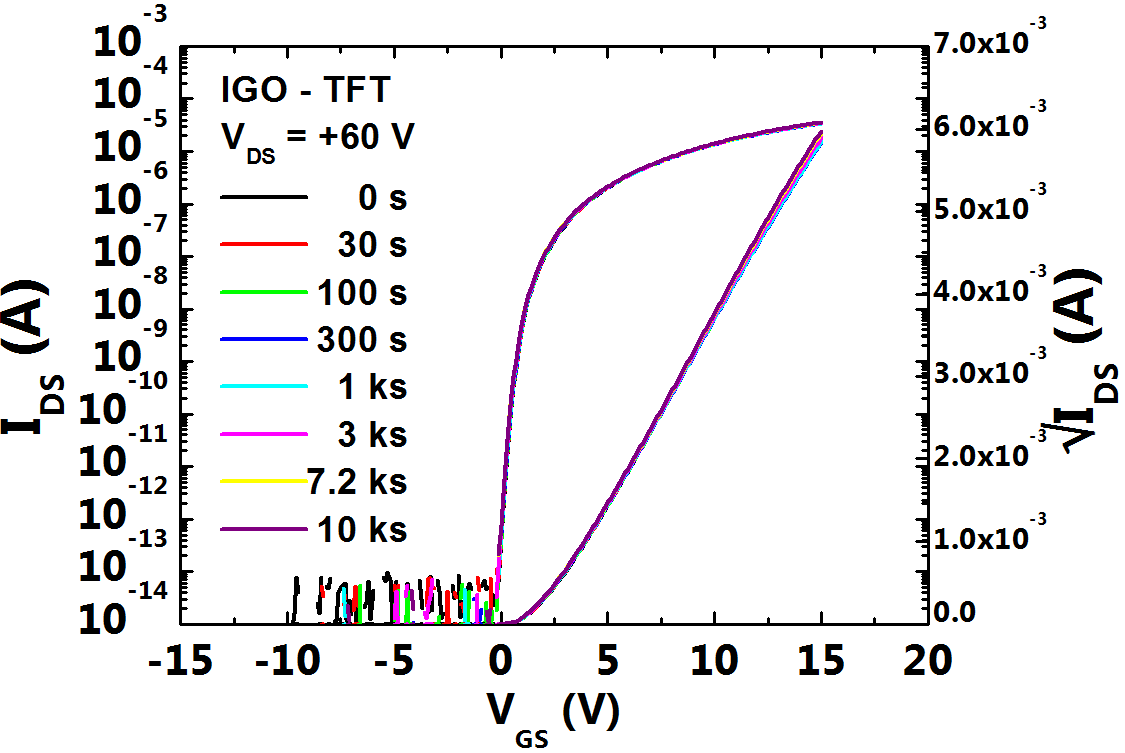


S6-(e)

**Figure S6.** Variation in the I_DS_−V_GS_ transfer characteristics for the IGZO TFT under the various DBS conditions of (a) 10, (b) 20, and (c) 40 V and IGO TFT under the DBS condition of (d) 40 V and (e) 60 V with the lapse of stress time for 10^4^ s. V_DS_ of 10 V was applied for the measurements.

**
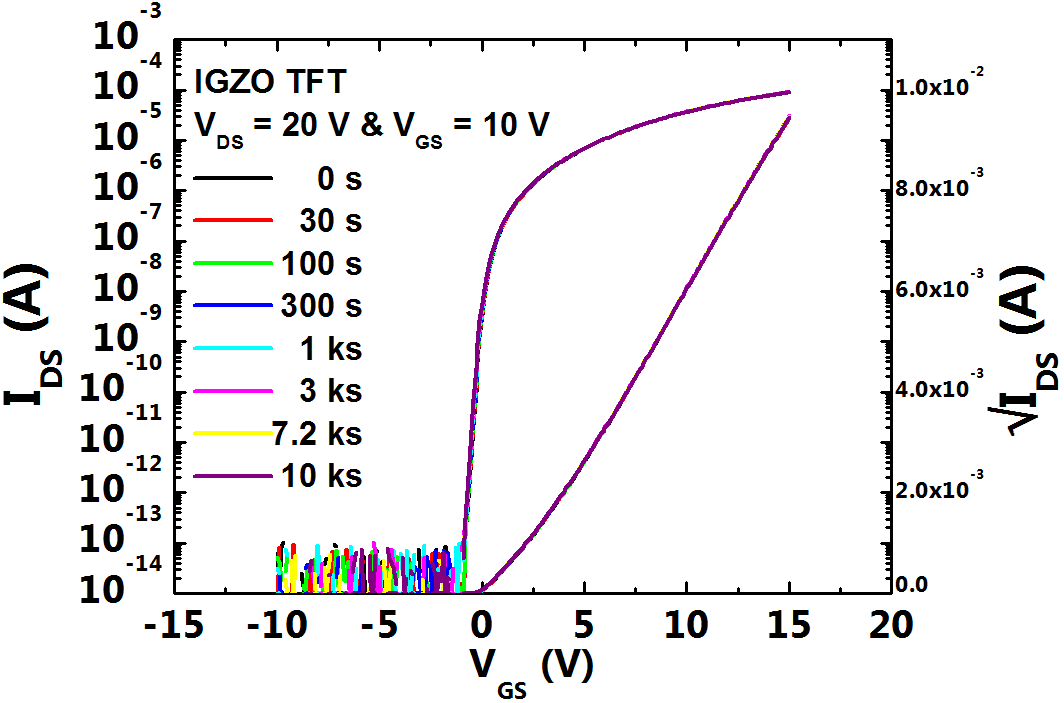
**

**Figure S7.** Variation in the I_DG_−V_GS_ transfer characteristics for the IGZO TFT with a lapse of stress time for 10^4^ s under the DBS of 20 V along with positive gate bias stress (PBS) of 10 V. A V_DS_ of 10 V was applied for the measurements.
